# Evidence that remodeling of insular cortex neurovascular unit contributes to hypertension‐related sympathoexcitation

**DOI:** 10.14814/phy2.13156

**Published:** 2017-03-07

**Authors:** Fernanda R. Marins, Jennifer A. Iddings, Marco A. P. Fontes, Jessica A. Filosa

**Affiliations:** ^1^ Departamento de Fisiologia e BiofísicaINCT, Instituto de Ciências BiológicasUniversidade Federal de Minas GeraisBelo HorizonteMinas GeraisBrazil; ^2^ Department of PhysiologyAugusta UniversityAugustaGeorgia

**Keywords:** Hypertension, Insular cortex (IC), neurovascular unit (NVU), NMDA receptors

## Abstract

The intermediate region of the posterior insular cortex (intermediate IC) mediates sympathoexcitatory responses to the heart and kidneys. Previous studies support hypertension‐evoked changes to the structure and function of neurons, blood vessels, astrocytes and microglia, disrupting the organization of the neurovascular unit (NVU). In this study, we evaluated the functional and anatomical integrity of the NVU at the intermediate IC in the spontaneously hypertensive rat (SHR) and its control the Wistar‐Kyoto (WKY). Under urethane anesthesia, NMDA microinjection (0.2 mmol/L/100 nL) was performed at the intermediate IC with simultaneous recording of renal sympathetic nerve activity (RSNA), heart rate (HR) and mean arterial pressure (MAP). Alterations in NVU structure were investigated by immunofluorescence for NMDA receptors (NR1), blood vessels (70 kDa FITC‐dextran), astrocytes (GFAP), and microglia (Iba1). Injections of NMDA into intermediate IC of SHR evoked higher amplitude responses of RSNA, MAP, and HR. On the other hand, NMDA receptor blockade decreased baseline RSNA, MAP and HR in SHR, with no changes in WKY. Immunofluorescence data from SHR intermediate IC showed increased NMDA receptor density, contributing to the SHR enhanced sympathetic responses, and increased in vascular density (increased number of branches and endpoints, reduced average branch length), suggesting angiogenesis. Additionally, IC from SHR presented increased GFAP immunoreactivity and contact between astrocyte processes and blood vessels. In SHR, IC microglia skeleton analysis supports their activation (reduced number of branches, junctions, endpoints and process length), suggesting an inflammatory process in this region. These findings indicate that neurogenic hypertension in SHR is accompanied by marked alterations to the NVU within the IC and enhanced NMDA‐mediated sympathoexcitatory responses likely contributors of the maintenance of hypertension.

## Introduction

Essential (or “neurogenic”) hypertension is a major health risk factor (Faraci et al. 1990; Mozaffarian et al. 2015) and key contributor to cognitive decline (Sabbatini et al. 2002; Iadecola and Davisson 2008) and stroke (Johansson 1999). However, the central mechanisms underlying hypertension are poorly understood (Ichihara 2015; Mozaffarian et al. 2015; Biancardi and Stern 2016). Studies have shown that cortical regions including the medial prefrontal cortex (Muller‐Ribeiro et al. 2012), anterior cingulate cortex (Cechetto and Chen 1990) and the insular cortex (Cechetto and Saper 1987; Oppenheimer 2007) are involved in the maintenance and regulation of heart rate, blood pressure and autonomic control and thus may play a role in the development of hypertension (Benarroch 1993; Asahina 2012; Marins et al. 2016). To this end, the insular cortex (IC), a key component of the central autonomic network, has been implicated in cardiovascular and autonomic control under physiological (Yasui et al. 1991; Oppenheimer 2007; Nagai et al. 2010; Alves et al. 2011; Chopra et al. 2011; Marins et al. 2016) and pathological conditions, including hypertension (Butcher and Cechetto 1995a, 1995b; Laowattana et al. 2006; Nagai et al. 2009; Kobayashi et al. 2012; de Morree et al. 2016). In humans, the IC has been shown to be involved in sympathoexcitatory responses following stroke and in intraoperative insular stimulation prior to temporal lobectomy for seizure control in epileptic patients (Oppenheimer et al. 1992a; Zhang et al. 1999; Laowattana et al. 2006).

Hypertensive patients subjected to proton magnetic resonance spectroscopy present alterations in metabolism associated with myelin damage (Ben Salem et al. 2008) in the thalami and IC when compared to normotensive controls. In the same way, during transient hypertension produced by norepinephrine, Sprague‐Dawley rats subjected to functional magnetic resonance imaging, show increased IC activation (Wang et al. 2006). An increase in cytochrome oxidase in the posterior insular cortex in spontaneously hypertensive mice (SHM) has also been reported, suggesting that in hypertension, increased metabolism and/or a mitochondrial dysfunction in this region could result in overactivation of the sympathetic nervous system (Strazielle et al. 2004). Therefore, while previous studies support a role for IC in hypertension (Strazielle et al. 2004; Wang et al. 2006), the mechanisms underlying this phenomenon are largely unknown.

Because hypertension is multifactorial, understanding its effects on multiple cell types may provide a better framework of the mechanisms involved. The neurovascular unit (NVU) is composed of multiple cell types including vascular cells, astrocytes, microglia, and neurons (Filosa 2010). How the complex interactions between these various cell types are affected in different brain regions, including the IC, under disease conditions is not clear (Zonta et al. 2003; Lok et al. 2007). Previous evidence indicates that hypertension alters the structure and function of blood vessels, astrocytes and microglia and disrupts the architecture of the NVU within the paraventricular nucleus of the hypothalamus, frontal cortex, occipital cortex, striatum, and hippocampus (Tomassoni et al. 2004a; Biancardi and Stern 2016). Hypertension‐induced vascular changes include rarefaction (Sokolova et al. 1985; Paiardi et al. 2009), remodeling (Mulvany 2012; Pires 2013), endothelial cell damage (Didion and Faraci 2003; Yamakawa et al. 2003), and changes in blood‐brain barrier permeability (Mayhan et al. 1986; Pires 2013). Reported vascular changes are associated with astrocyte and microglia alterations including: increased expression of glial fibrillary acidic protein (GFAP)(Tomassoni et al. 2004a), increased endothelial cell permeability (Tagami et al. 1990), microglial activation manifested by de‐ramified morphology and proinflammatory cytokine upregulation (Morrison and Filosa 2013; Shen et al. 2015; Biancardi and Stern 2016). Changes in glial structure and function are likely contributors to neuroinflammation and increased neuronal excitation, thus increasing the activity of pathways contributing to elevated blood pressure. However, whether hypertension causes remodeling of the NVU in the IC is not known.

We recently described a functional topography for autonomic and cardiovascular regulation in the posterior IC (Buffo et al. 2010). Specifically, NMDA receptor (NMDAR) expression is present within the intermediate region of the posterior IC (intermediate IC), and NMDA receptor stimulation within this region results in sympathoexcitatory responses in the heart and kidneys (Buffo et al. 2010). Thus, the goal of this study was to evaluate the integrity of the NVU in the intermediate IC and the contribution of NMDA‐evoked sympathoexcitatory responses in this region to neurogenic hypertension. Using in vivo and in situ studies, we evaluated NMDA receptor activation‐ or inhibition‐evoked cardiovascular responses and changes to the NVU within the IC sympathoexcitatory region in spontaneously hypertensive rats.

## Methods

### Animals

All experiments were performed in male aged‐match Wistar Kyoto (WKY) and spontaneously hypertensive rats (SHR). In SHR, arterial blood pressure peaks between 12 and 16 weeks of age (Tanase et al. 1982); thus, all studies were conducted in 14 week old animals. For functional experiments, performed at Federal University of Minas Gerais (UFMG), rats were bred at the animal facilities of the Biological Sciences Institute (CEBIO, UFMG) and conducted in accordance with the guidelines established by CETEA/UFMG (protocol 11412/2012). For immunofluorescence experiments, performed at Augusta University, rats were purchased from Harlan Laboratories (IN, USA); all experiments were conducted in accordance to NIH guidelines and carried out in agreement with Augusta University Institutional Animal Care and Use Committee Guidelines. All animals were housed at the institutional facilities with a 12 h light/dark cycle and ad libitum access to food and water.

### NMDA and NMDA receptor antagonist (D‐2‐amino‐5‐phosphonopentanoate, AP‐5) microinjection into intermediate Insular Cortex

For functional experiments rats were anesthetized with urethane (1.4 g/kg i.p.), and the trachea cannulated to maintain an open airway. Body temperature was maintained at 37–37.5°C using a heating pad (Physitemp TCAT‐2DF Controller). The animal was positioned in a stereotaxic frame (Lab Standard with 18 Degree Ear bars, Stoelting, IL, USA), and a small unilateral, in NMDA microinjection, or bilateral, in AP‐5 microinjection, craniotomy was made to allow insertion of a glass pipette (Sigma‐Aldrich) into the intermediate IC: AP +0.0 mm from bregma, 5.8 mm lateral and 7.0 mm ventral (Marins et al. 2016). Catheters were placed into the femoral artery to record mean arterial pressure (MAP) and heart rate (HR), and into the vein for anesthetic supply if necessary. Using a retroperitoneal approach, the left renal nerve was isolated and prepared for renal sympathetic nerve activity (RSNA) recordings as previously reported (Silva et al. 2005). Following surgical procedures and stabilization of cardiovascular parameters, NMDA (0.2 mmol/L/100 nL, Sigma Chemical, St. Louis, MO) or NMDA receptor antagonist D‐2‐amino‐5‐phosphonopentanoate (AP‐5, 5 mmol/L/100 nL, Sigma Chemical, St. Louis, MO) microinjections were made in the intermediate IC (sympathoexcitatory cardiovascular zone) of WKY and SHR animals (*n* = 4 each group NMDA microinjection and *n* = 6 each group AP‐5 microinjection, separated groups). As a control of the cardiovascular responses specificity, we performed NMDA or AP‐5 microinjections in the anterior IC: AP +3.72 mm from bregma, 5.4 mm lateral and 6.8 mm ventral (*n* = 4, each group). NMDA microinjection outside the insular cortex (*n* = 3 in each coordinate) was included as a negative control group. At the end of each experiment, after urethane overdose to record RSNA noise, a 100 nL microinjection of evans blue were performed and the animals were transcardially perfused. The brain was dissected and maintained in 4% PFA for 4 h and then transferred to a 30% sucrose solution for 48 h. Insular cortex sections from intermediate (0.0 mm) insular cortex were collected and then processed for analysis in a microscope. Microinjection site in the IC was confirmed using the atlas of Paxinos and Watson (1986) as a reference.

### Immunofluorescence

#### Paraformaldehyde (PFA) brain perfusion

WKY and SHR were deeply anesthetized with sodium pentobarbital (100 mg/kg, i.p.) followed by transcardial perfusion with 0.01 mol/L phosphate‐buffered saline (PBS, 150 mL) and 4% PFA (350 mL). Brains were dissected and postfixed 4 h in 4% PFA followed by cryoprotection in PBS containing 30% sucrose for 3 days at 4°C. 50 *μ*m sections containing the anterior and intermediate IC were collected.

#### FITC dextran (70 kDa) perfusion to mark the cerebral microvasculature

WKY and SHR were anesthetized with a ketamine/xylazine cocktail (60 mg and 8 mg/mL, respectively, intramuscular injection), and 70 kilodalton (kDa) fluorescein isothiocyanate (FITC)‐dextran (50 mg/mL dissolved in sterile 0.9% saline; 1.43 *μ*L/g) was administered via intrajugular injection. After 10 min of FITC‐dextran circulation, the brain was removed and fixed in 4% PFD for 48 h at 4°C, followed by cryoprotection in PBS containing 30% sucrose for 3 days at 4°C. 50 *μ*m sections containing the anterior and intermediate IC were collected.

#### Targeted areas

The rostrocaudal distribution of the IC is extensive and involves different functional aspects. For this reason, the selected area of the intermediate IC was localized between granular and dysgranular layers at anteroposterior level 0.0 mm (bregma level). This area is known to be involved with autonomic cardiovascular control (Cechetto 1987; Butcher and Cechetto 1995b; Carmeliet 2005; Attwell et al. 2010) and where NMDA microinjection elicited sympathoexcitatory responses (Attwell et al. 2010). For control experiments, the anterior agranular IC (anterior IC; +3.72 mm anterior to bregma) was chosen. This is a noncardiovascular area of the IC that is involved in pain (Hu et al. 2015), taste (Dalenberg et al. 2015), emotional awareness (Simmons et al. 2013; Feinstein et al. 2016) and fear (Lipp et al. 2015).

#### Antibodies

Fixed slices were blocked for 1 h in 0.01 mol/L PBS containing 0.3% Triton X‐100, 0.04% NaN_3_ and 10% horse serum (Vector Labs, Burlingame, CA), followed by primary antibody incubation. For PFA perfused slices primary antibodies included: NR1 (anti‐goat NMDA zeta 1‐polyclonal, diluted 1:100; Santa Cruz Biotechnology, CA, sc‐1467 Lot#J3012.) and NeuN (anti‐mouse neuronal nuclei marker, diluted 1:1000; Milipore, CA, MAB377) incubated for 48 h at room temperature and revealed by secondary reaction with anti‐goat FITC and anti‐mouse Cy3, respectively (1:250, Jackson Immunoresearch, West Grove, PA); slices were counterstained with TOTO‐3 iodide (1:1000, Invitrogen, 5 min of incubation) as previously described (Biancardi et al. 2010). For dextran perfused, brains primary antibodies included: GFAP (anti‐mouse glial fibrillary acidic protein, diluted 1:1000; Chemicon, CA, AB5804) and Iba1 (anti‐rabbit ionized calcium‐binding adapter molecule 1, diluted 1:1000; Wako, VA, catalog number: 019‐19741) incubated overnight at room temperature and revealed by secondary reaction with anti‐mouse Cy5 (1:50) and anti‐rabbit Cy3 (1:250), respectively (Jackson Immunoresearch, West Grove, PA, USA). All antibodies were diluted with PBS containing 0.3% Triton X‐100 and 0.4% NaN_3_; sections were mounted with Vectashield (Vector laboratories, CA, USA). The specificity of each antibody was tested by the omission of the primary antibody.

#### Confocal image acquisition

Images of stained sections were acquired using a Zeiss LSM510 confocal scanning microscope (Carl Zeiss, GER) as previously described (Biancardi et al. 2010; Iddings et al. 2015). Images from consecutive optical focal planes (1 *μ*m interval), were taken using a 25× or 40× oil immersion objective, and a projection image of the sections was generated. For NMDAR (*n* = 6, per group), FITC dextran (*n* = 13, per group), Iba1 (*n* = 13, per group) and GFAP (*n* = 13, per group) 30 *μ*m consecutives images were acquired at 40×. Additionally, for FITC dextran (*n* = 10, per group) 50 *μ*m consecutive images were acquired at 25×. Images from both hemispheres of WKY and SHR groups were acquired by Zen acquisition software (Carl Zeiss Microscopy, Oberkochen, Germany) and digitized with identical acquisition settings for further comparison.

#### Immunofluorescence analysis

NMDAR, vascular density, GFAP and Iba1 expression were expressed as percent area using ImageJ software (NIH). The maximum projection image was thresholded, converted to binary and analyzed using Image J histogram. For blood vessels and microglia, images were skeletonized before histogram analysis (Morrison and Filosa 2013). Skeletonize analysis parameters used to determine angiogenesis and microglia morphological changes included: branch number, branch length, junction number, and endpoint number (Streit 2000; Morrison and Filosa 2013; Shen et al. 2015). The contact between astrocytes and blood vessels was also analyzed using the Image J colocalization plug‐in.

### Statistical analysis

Data were expressed as mean ± SEM. “n” represents the number of microinjections or regions imaged in each experiment. Differences between means from each group (WKY vs. SHR) were evaluated using unpaired Student's t test. Single within‐group comparisons with control or baseline were evaluated using a paired Student's t test. Multiple group comparisons were evaluated using one‐way ANOVA with Bonferroni multiple comparison post‐test. *P *<* *0.05 was considered significant for all analyses.

## Results

### Cardiovascular responses evoked by NMDA receptor activation and blockade in the intermediate IC

**Figure 1:**
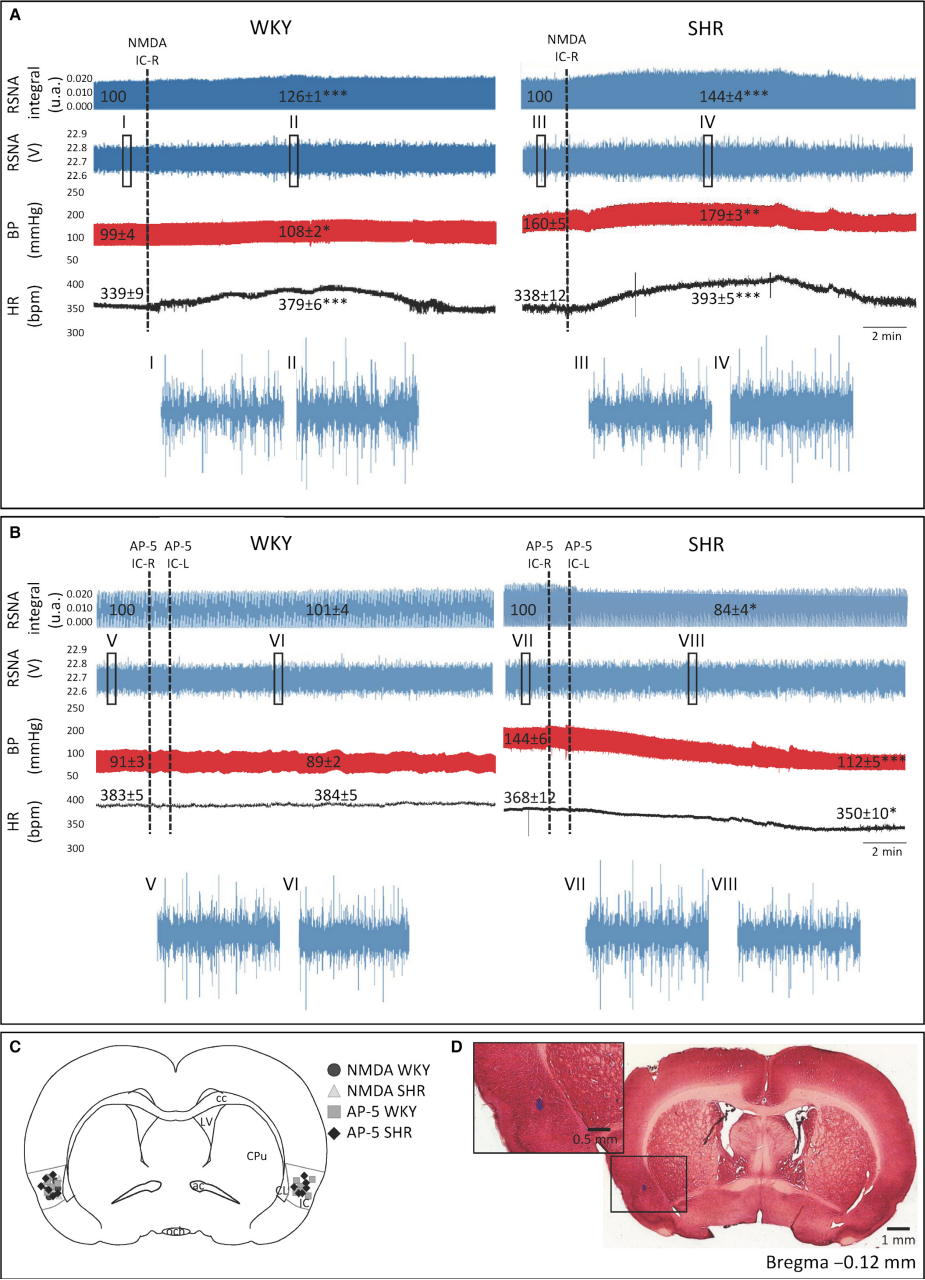
Representative tracings showing the effect of NMDA and AP‐5 microinjection into intermediate IC. Representative tracings showing changes on cardiovascular and autonomic baseline parameters (HR, heart rate in bpm; MAP, mean arterial pressure in mmHg; RSNA, renal sympathetic nerve activity in % of the baseline) elicited by unilateral NMDA (0.2 mM,* n* = 4) microinjection **(A)** or by bilateral AP‐5 (5 mM,* n* = 6) microinjection **(B)** into intermediate IC of WKY (panel on the left) and SHR (panel on the right). The numbers near the traces are the mean values for the predrug and mean maximum value for postdrug conditions for the respective group. I‐VIII show representatives tracings of RSNA discharges (a.u.). Statistical differences between predrug and postdrug tested using Paired T test, *P < 0.05, **P < 0.01 and ***P < 0.001 versus predrug values. All data are mean ± SEM. The schematic coronal sections of the rat brain corresponding to the atlas of Paxinos and Watson (1986) illustrating sites of injections into the intermediate IC, circle correspond to NMDA microinjection in WKY, triangle correspond to NMDA microinjection in SHR, square correspond to AP‐5 microinjection in WKY, diamond correspond to AP‐5 microinjection in SHR **(C)**. A representative photomicrograph shows a microinjection site into the right IC at intermediate level **(D)** where microinjection evoked cardiovascular and autonomic responses. Corpus callosum (cc); lateral ventricle (LV); caudate putamen (CPu); claustrum (CL); insular cortex (IC); anterior commissure (ac); optic chiasm (och).

**Figure 2:**
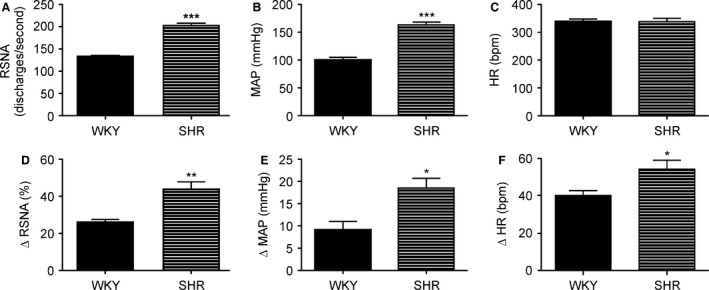
Changes in autonomic and cardiovascular responses evoked by NMDA or AP‐5 microinjection at intermediate IC of SHR. Baseline sympathetic nerve activity discharge (**A**, RSNA, discharges/second), mean arterial pressure (**B**, MAP, mmHg) and heart rate (**C**, HR, bpm) of WKY (black bars) and SHR (black vertical stripes) measured 15 minutes before any microinjection (*n* = 10). Changes in RSNA (**D**, %), MAP (**E, mmHg**) and HR (**F, bpm**) in WKY (black bars) and SHR (black vertical stripes) evoked by unilateral NMDA microinjection (0.2 mM) within the intermediate IC (coordinates AP: 0.0 mm, LL ‐5.6 mm and DV: ‐7.0 mm, *n* = 4 each group). Effect of by bilateral AP‐5 microinjection (5 mM) within the intermediate IC (coordinates AP: 0.0 mm, LL ‐5.6 mm and DV: ‐7.0 mm, *n* = 6 each group) on RSNA (**G**, %), MAP (**H, mmHg**) and HR (**I, bpm**) in WKY (black bars) and SHR (black vertical stripes). Statistical differences between WKY and SHR tested using Unpaired T test, * *P* < 0.05, ** *P* < 0.01 and *** *P* < 0.001. All data are mean ± SEM.

To evaluate possible hypertension‐evoked changes in NMDA receptor responses within the intermediate IC, we compared the effect of NMDA or AP‐5 microinjection into the intermediate IC on RSNA, MAP and HR in urethane anesthetized WKY and SHR (*n* = 4, each group NMDA microinjection and *n* = 6, each group AP‐5 microinjection). Representative tracings showing effects on baseline cardiovascular and autonomic parameters (HR, MAP, and RSNA) elicited by NMDA microinjection into the unilateral intermediate IC (Fig. 1A) or AP‐5 microinjection into the bilateral intermediate IC (Fig. 1B) of WKY or SHR are shown in Figure 1. SHR displayed a significantly higher resting baseline (*n* = 10) discharge of RSNA (SHR 196.7 ± 4.87 vs. WKY 130.5 ± 1.86 discharges/second, *P *<* *0.0001) and MAP (SHR 160.3 ± 5.33 vs. WKY 99 ± 4.02 mmHg, *P *<* *0.0001). Heart rate did not differ between groups (Fig. 2A–C). Unilateral NMDA microinjection into the intermediate IC of SHR evoked larger amplitude responses of RSNA amplitude (∆=SHR 43.7 ± 4.09 vs. WKY 26 ± 1.5% of baseline, *P *=* *0.0066), MAP (∆=SHR 18.53 ± 2.2 vs. WKY 9.14 ± 1.84 mmHg, *P *=* *0.0171), and HR (∆=SHR 54.28 ± 4.88 vs. WKY 40.32 ± 2.55 bpm, *P *=* *0.0446) when compared to WKY (Fig. 2D–F). Bilateral AP‐5 microinjections into the intermediate IC of SHR evoked a decrease in RSNA amplitude (∆=SHR ‐16 ± 3.6 vs. WKY 1 ± 4.1% of baseline, *P *=* *0.0204), MAP (∆=SHR −31.7 ± 4.8 vs. WKY −1.3 ± 1.4 mmHg, *P *<* *0.0001), and HR (∆=SHR −18.16 ± 9.5 vs. WKY 0.6 ± 0.8 bpm, *P *=* *0.0381) when compared to WKY (Fig. 2G–I). Histological analysis performed at the end of the experiment confirmed the location of the microinjection site, specific to the intermediate region of the posterior IC between the granular and dysgranular areas (Fig. 1C–D). To assess the specificity of cardiovascular responses, we performed NMDA or AP‐5 microinjections in the anterior IC (*n* = 4). Neither unilateral microinjection of NMDA (RSNA: ∆=SHR +5 ± 4.02 vs. WKY −6 ± 3.45% of baseline, *P *=* *0.332, MAP ∆=SHR 1.92 ± 2.45 vs. WKY 4.34 ± 2.64 mmHg, *P *=* *0.5896 and HR ∆=SHR 4.66 ± 3.64 vs. WKY −4.92 ± 3.95 bpm, *P *=* *0. 1761) nor bilateral microinjection of AP‐5 (RSNA: ∆=SHR 8.3 ± 5.15 vs. WKY ‐6 ± 5.73% of baseline, *P *=* *0.2537, MAP ∆=SHR 0.18 ± 3.6 vs. WKY 2.67 ± 1.96 mmHg, *P *=* *0.459 and HR ∆=SHR 6.45 ± 4.23 vs. WKY 5.87 ± 3.76 bpm, *P *=* *0.4895) evoked changes in autonomic and cardiovascular baseline parameters. Histological analysis performed at the end of the experiment confirmed the location of the microinjection site in the anterior agranular IC. In addition, microinjection of NMDA or AP‐5 outside of the insular cortex anatomical limits (peri‐insular regions, *n* = 3 each group) in WKY or SHR did not evoke significant changes in cardiovascular parameters (mean maximum changes of NMDA WKY: Δ HR: 2 ± 2 bpm, Δ MAP: 2 ± 1 mmHg, Δ RSNA: 2 ± 1% and NMDA SHR: Δ HR:−3 ± 2 bpm, Δ MAP: 1 ± 1 mmHg, Δ RSNA: 4 ± 1%; AP‐5 WKY: Δ HR: 7 ± 4 bpm, Δ MAP: 4 ± 2 mmHg, Δ RSNA: 8 ± 6% and AP‐5 SHR: Δ HR: 5 ± 2 bpm, Δ MAP: 0 ± 4 mmHg, Δ RSNA: 10 ± 5%).

### NMDA receptor density in the intermediate IC

**Figure 3:**
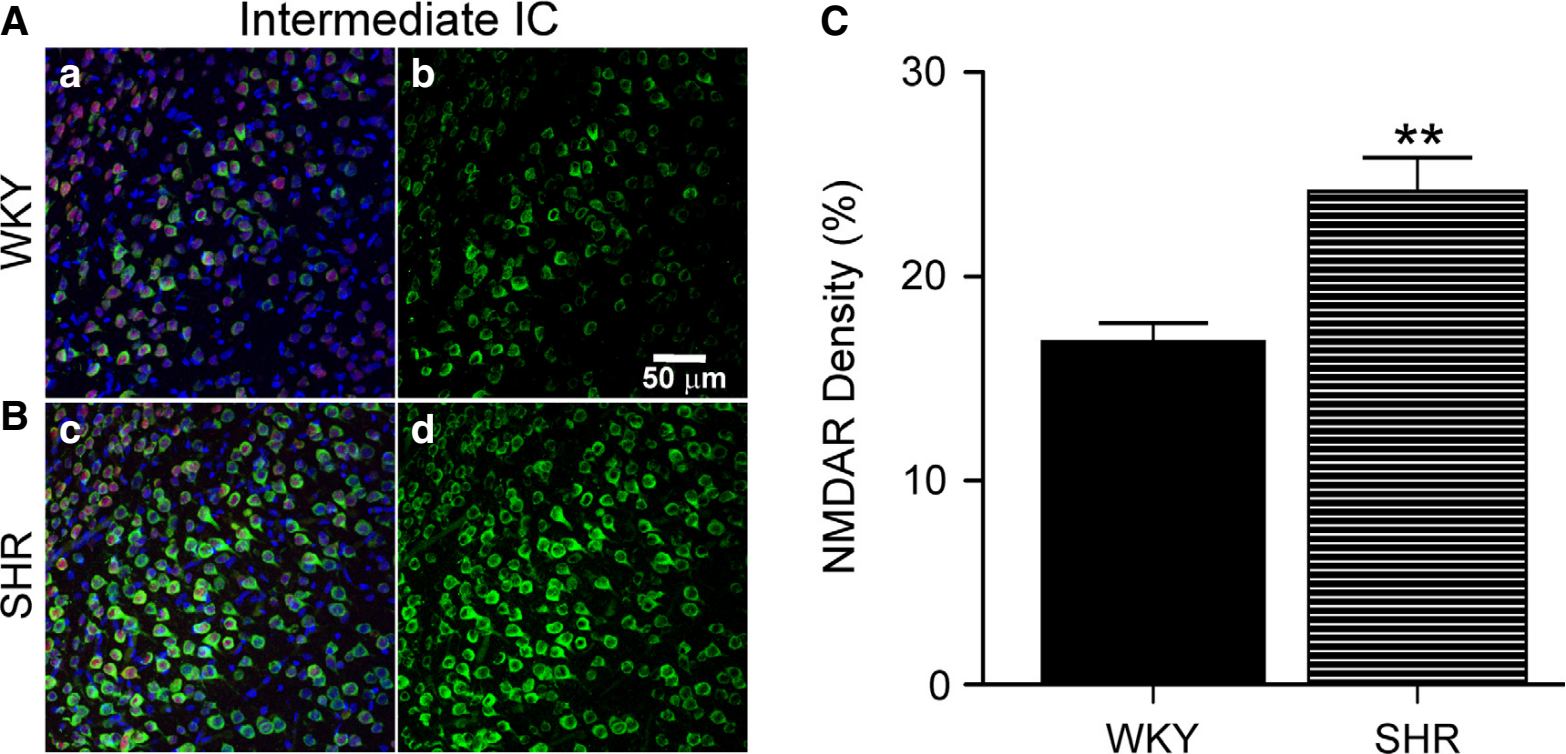
Increased NMDAR density in intermediate IC of SHR. (**A**‐**B**) Representative immunofluorescence confocal images from intermediate IC (coordinates AP 0.0 mm, LL ‐5.6 mm and DV: ‐7.0 mm) of WKY (**a**‐**b**) and SHR (**c**‐**d**) stained for NMDA receptor (NMDAR; green) NR1 subunit, NeuN (red), and counterstained with the nuclear marker TOTO‐3 (blue) (**a** and **c**). The neuronal marker NeuN (red) and the nuclear marker TOTO (blue) were used as counterstains to identify the cortical region imaged during acquisition. Representative image of NMDAR (green) maximal intensity (**b** and **d**) was the quantified image. All images were acquired at 40× magnification. (**C**) Mean increase in percent NMDAR density in WKY and in SHR (*n* = 6 each group). Statistical differences between WKY and SHR tested using Unpaired T test, ** *P* < 0.01, mean ± SEM. Scale bar = 50 *μ*m.

Animals used for immunofluorescence protocols had their mean systolic blood pressure measured via tail cuff plethysmography. Baseline values for mean systolic blood pressure were 120.5 ± 1.96 mmHg and 183.1 ± 2.05 mmHg (*P *<* *0.0001) for WKY and SHR, respectively. Considering the possibility that augmented cardiovascular responses evoked by NMDA receptors stimulation in the intermediate IC of SHR resulted from changes in NMDA receptor (NMDAR) expression, we performed immunofluorescence of NMDAR NR1 subunit in this region. As shown in Figure 3, NR1 density was significantly increased in SHR compared to WKY (24.17 ± 1.68% vs. 16.67 ± 1.05%, *n* = 6 each group, *P *=* *0.0036).

### Vascular density within intermediate IC

**Figure 4:**
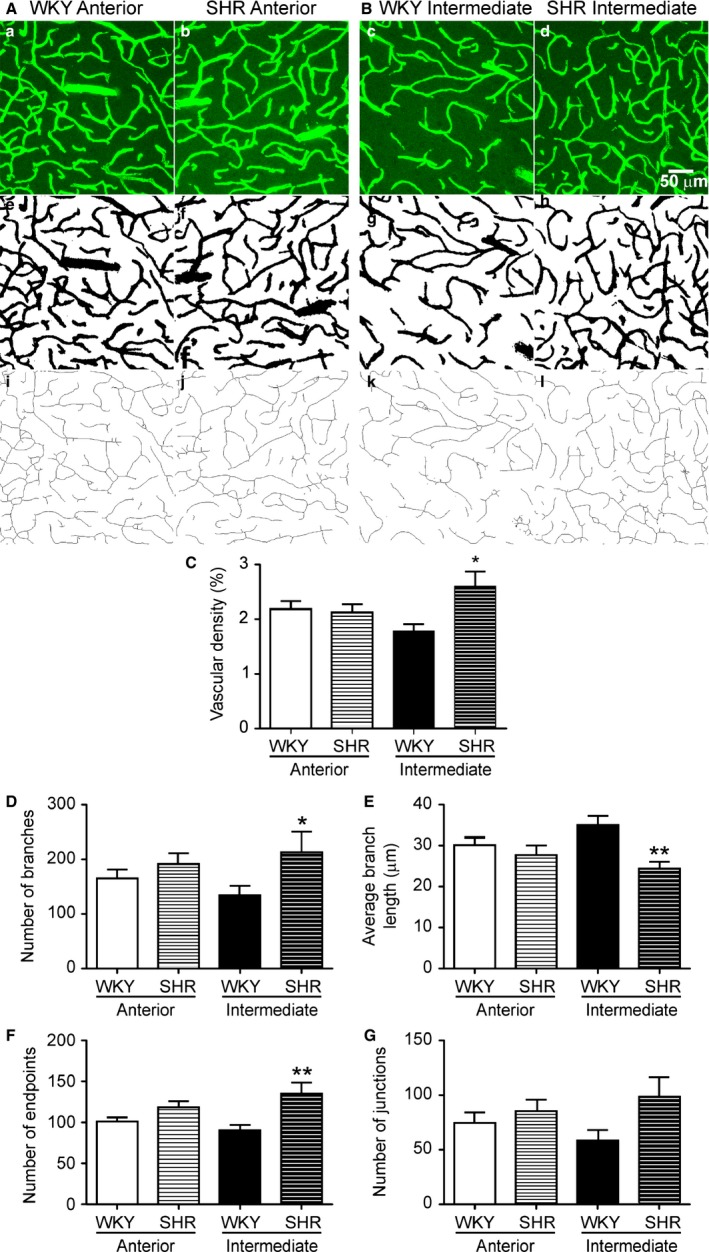
Increased angiogenesis in SHR intermediate IC. Representative confocal maximum intensity projection **(a‐d)**, binary **(e‐h)** and skeletonized **(i‐l)** images of WKY and SHR vasculature in **(A)** anterior and **(B)** intermediate IC marked by intravascular injection with 70 kilodalton fluorescein isothiocyanate dextran (FITC‐70) (green). Skeleton analysis mean data showing: percent vascular density (**C**), number of branches **(D),** average branch length **(E)**, number of endpoints **(F)** and number of junctions **(G)** in the anterior and intermediate IC of SHR and WKY. All images were acquired at 25× magnification. Statistical differences between the same regions of WKY and SHR tested using one‐way ANOVA with Bonferroni multiple comparison post‐test (*n* = 10 each group, * *P* < 0.05, ** *P* < 0.01). All data are mean ± SEM. Scale bar = 50 *μ*m.

To evaluate possible changes in vascular morphology, we quantified vascular density in the anterior and intermediate IC of SHR and WKY. To determine if hypertension‐evoked morphological changes are restricted to the cardiovascular region (intermediate) of the IC, all immunofluorescence experiments were compared to the anterior IC, a noncardiovascular region. Figure 4A, B, shows representative confocal images (Fig. 4a–d), corresponding binary (Fig. 4e–h) and skeletonized images (Fig. 4i–l) of the anterior and intermediate IC for SHR and WKY. No differences in vascular density between SHR and WKY were found within the anterior region of the IC (*n* = 10 each group, *P *=* *0.79, Fig. 4C). However, within the SHR intermediate region, a significant increase in vascular density was observed (*n* = 10 each group, *P *=* *0.016, Fig. 4C). Supporting angiogenesis within the intermediate IC of SHR, we observed a significant increase in the number of branches (*n* = 10 each group, *P* = 0.0481, Fig. 4D), reduction in average branch length (*n* = 10 each group, *P* = 0.0053, Fig. 4E) and increased number of endpoint voxels (*n* = 10 each group, *P* = 0.0043, Fig. 4F). No significant changes in the number of junctions were found (Fig. 4G).

### GFAP expression in astrocytes and cell contact with blood vessels in the intermediate IC

**Figure 5:**
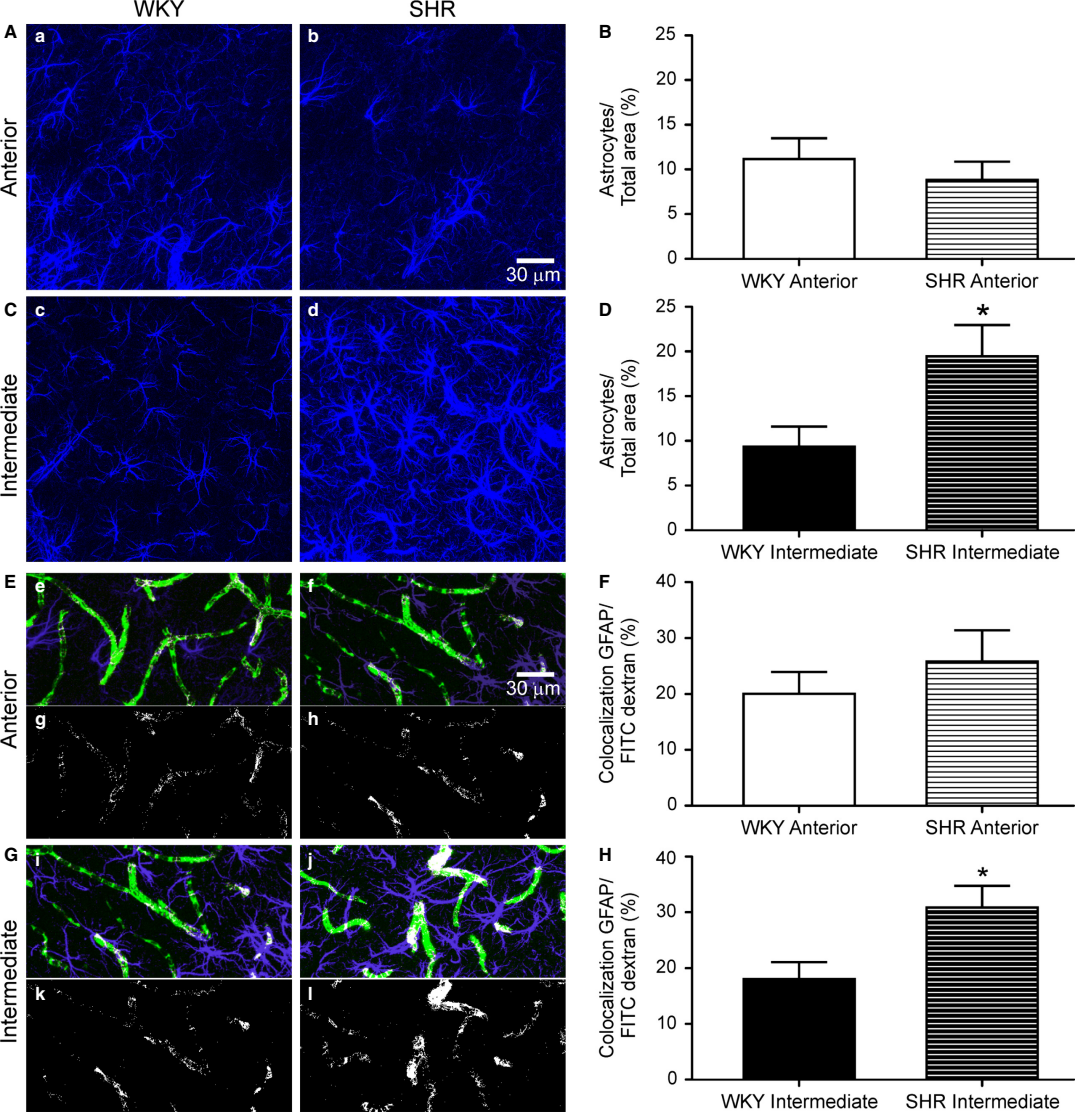
Astrocyte density and astrocyte‐vascular colocalization are increased in SHR intermediate IC. Representative confocal maximum intensity projection images of astrocytes at **(A)** anterior and **(C)** intermediate IC of WKY and SHR. Summary data showing mean GFAP (anti‐mouse glial fibrillary acidic protein,) density **in** anterior IC (B) and intermediate IC (**D**) of WKY and SHR. Colocalization between vasculature (FITC‐70) and GFAP are demonstrated in **(E)** for anterior IC and in **(G)** for intermediate IC. Representative images of colocalization (white) between astrocytes (blue) and vasculature (green) in anterior IC **(E, e‐f)** and intermediate IC **(G, i‐j)**.Representative images of colocalized points (8‐bit image) between vasculature and GFAP in anterior IC **(E, g‐h)** and intermediate IC **(G, k‐l)**. Summary data showing mean GFAP FITC dextran colocalization in anterior IC (**F**), and intermediate IC (**H**) of WKY and SHR. All images were acquired at 40× magnification. Statistical differences between the same regions of WKY and SHR tested using Unpaired Student's *t*‐test (*n* = 13 each group, * *P* < 0.05). All data are mean ± SEM. Scale bar = 30 *μ*m.

To determine whether hypertension evoked structural changes to IC astrocytes from WKY and SHR, we measured the percent area of GFAP, a specific astrocyte cytoskeletal protein marker, in the region. As demonstrated in Figure 5(A–B), no differences were observed in GFAP expression in the anterior IC between WKY and SHR (*n* = 13 each group, *P *=* *0.4535). However, increased GFAP immunoreactivity was observed in the intermediate IC of SHR (∆ = 10.14%, *n* = 13 each group, *P *=* *0.0236, Fig. 5C–D). No differences in anterior IC astrocyte process‐blood vessel contact were observed between WKY and SHR (*n* = 13 each group, *P* = 0.4023, Fig. 5E–F). Conversely, SHR presented a marked increase in the contact between astrocyte processes and blood vessels in the intermediate IC (∆= 12.81%, *n* = 13 each group, *P *=* *0.0155, Fig. 5G–H).

### Microglia activation within the intermediate IC

**Figure 6:**
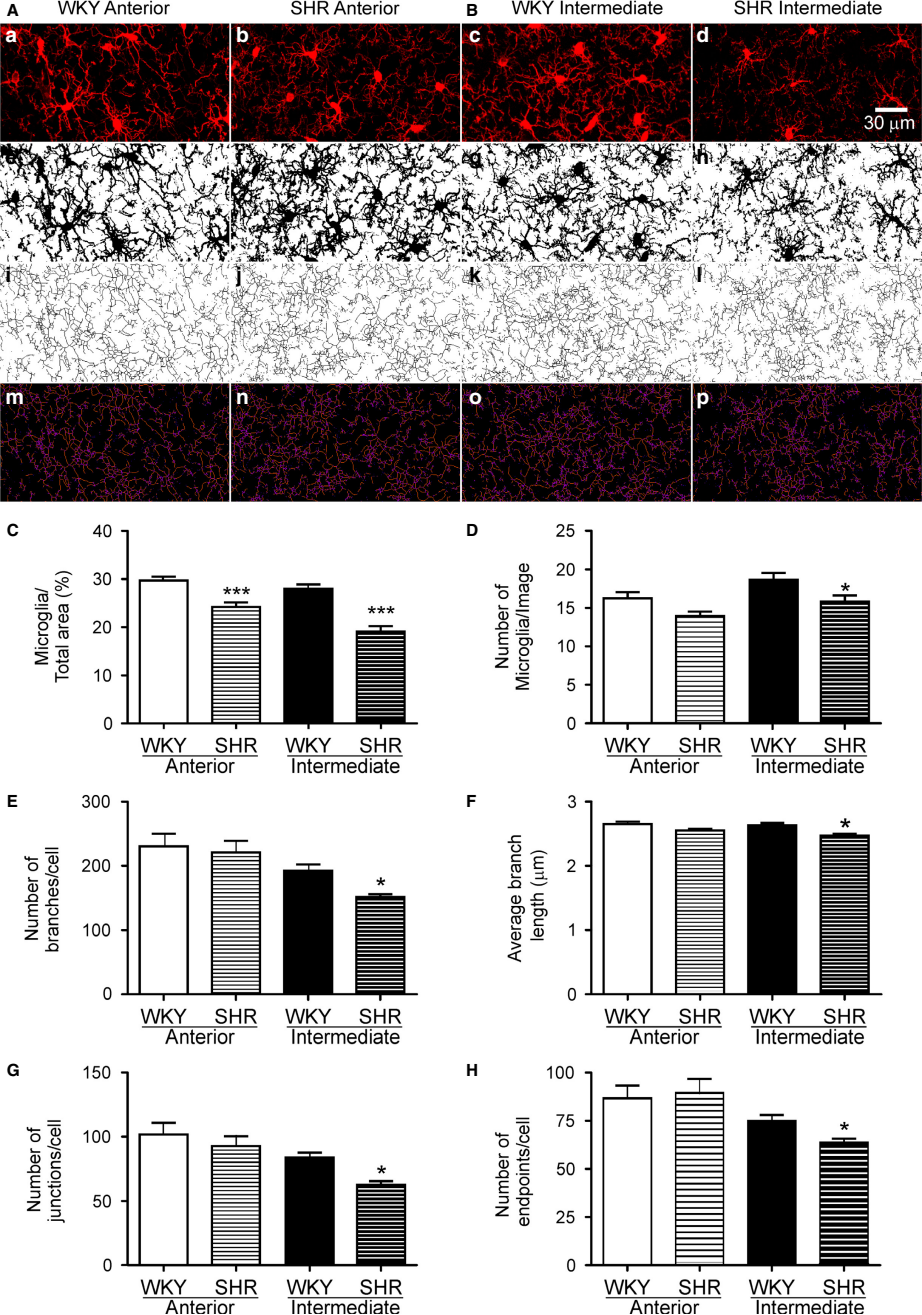
Reduced branch complexity and de‐ramified microglia morphology in SHR intermediate IC. Representative microglia images in anterior **(A)** and intermediate **(B)** IC of WKY and SHR (first row: confocal maximum intensity projection, second row: binary image, third row: skeleton image, fourth row: tagged skeleton). Summary data showing mean microglia percent area **(C)** number of microglia per image **(D)**, number of branches/cell **(E)**, average branch length (*μ*m) **(F)**, number of junctions/cell **(G)** and number of endpoints/cell **(H)**. All images were acquired at 40× magnification. Statistical differences between the same regions of WKY and SHR tested using one‐way ANOVA with Bonferroni multiple comparison post‐test (*n* = 10, each group, * *P* < 0.05, *** *P* < 0.001). All data are mean ± SEM. Scale bar = 30 *μ*m.

Microglial cells respond to brain injury and inflammation with a morphological change characterized by decreased process length (Streit 2000; Morrison and Filosa 2013). We measured microglia activation by measuring the degree of arborization as previously reported (Morrison and Filosa 2013). Representative confocal (*n* = 13 each group, each region) maximum intensity projection (Fig. 6a–d), binary (Fig. 6e–h), skeletonized (Fig. 6i–l) and skeleton plug‐in (Fig. 6m–p) images of anterior and intermediate IC are shown in Figure 6A–B. SHR showed reduced microglia density in both IC regions (Anterior: WKY 29.72 ± 0.77 vs. SHR 24.21 ± 0.9% of image area, *P *=* *0.0001 and Intermediate: WKY 27.99 ± 0.93 vs. SHR 19.13 ± 1.08% of image area, *P *<* *0.001, Fig. 6C). However, reduced microglia number was only observed in SHR intermediate IC (WKY 18.7 ± 0.86 vs. SHR 15.85 ± 0.76 microglia per image, *P *=* *0.0203, Fig. 6D). Moreover, the number of branches/cell (WKY 192.6 ± 9.47 vs. SHR 151.3 ± 4.79 number of branches/cell, *P *=* *0.0007, Fig. 6E), average microglia branch length (WKY 2.76 ± 0.04 vs. SHR 2.59 ± 0.03 *μ*m, *P *=* *0.0017, Fig. 6F), number of junctions/cell (WKY 82.92 ± 3.71 vs. SHR 61.97 ± 2.9, *P *=* *0.0002, Fig. 6G) and number of microglia process endpoints/cell (WKY 74.35 ± 3.07 vs. SHR 63.08 ± 2.11, *P *=* *0.0058, Fig. 6H) were also significantly reduced in the intermediate IC of SHR, without any differences observed in the anterior IC.

## Discussion

Limited studies have addressed functional and structural changes in the insular cortex during hypertension (Strazielle et al. 2004; Wang et al. 2006; Ben Salem et al. 2008). Ben Salem and colleagues reported decreased N‐acetylaspartate/creatine ratio in the IC of hypertensive humans using proton magnetic resonance spectroscopy (Ben Salem et al. 2008), suggesting alterations in IC metabolism. On the other hand, Wang and colleagues reported increased regional cerebral blood flow in the IC during transient hypertension in male Sprague‐Dawley rats using functional magnetic resonance imaging (Wang et al. 2006), indicating a IC activation during transient hypertension. In addition, Strazielle and colleagues observed increased cytochrome oxidase activity in the IC of spontaneous hypertensive mice (SHM) (Strazielle et al. 2004), possibly due to excitotoxicity and mitochondrial dysfunction. To our knowledge, this is the first study addressing the effects of hypertension on the NVU at a representative cardiovascular region of the IC. We report that the intermediate IC of SHR showed: *1‐* higher amplitude responses of RSNA, MAP, and HR to unilateral NMDA microinjections and a tonic effect of NMDAR in RSNA, blood pressure and HR control; *2‐* increased NMDAR density; *3‐* increased vascular density; *4‐* greater GFAP immunoreactivity; *5‐* increased contact between astrocyte processes and blood vessels and *6‐* enhanced microglia activation. Together, these data indicate that hypertension alters NMDA‐mediated sympathoexcitatory responses and significantly alters the structural organization of the NVU within the intermediate IC.

Previous studies demonstrated that the posterior insular cortex is involved in the control of autonomic and cardiovascular function under physiological (Oppenheimer and Cechetto 1990; Marins et al. 2016) and pathological (Oppenheimer 1992; Butcher and Cechetto 1995b; Laowattana et al. 2006; de Morree et al. 2016) conditions such as hypertension in rats (Butcher and Cechetto 1995a, 1995b), mice (Strazielle et al. 2004), and humans (Ben Salem et al. 2008; Nagai et al. 2009; Kobayashi et al. 2012). Supporting our findings of overactivation in intermediate IC of SHR and the tonic effect of this region in autonomic and cardiovascular control, in a series of elegant studies, Butcher and Cechetto (Butcher and Cechetto 1995b) showed that IC lesion (using the excitotoxin D,L‐homocysteic acid) significantly decreased MAP and RSNA, suggesting a tonic effect of the IC in blood pressure regulation in SHR. In agreement with our findings, showing that microinjection of NMDA or AP‐5 into the anterior IC or into the peri‐insular region, noncardiovascular regions, did not evoked any changes in evaluated autonomic and cardiovascular parameters, previous studies demonstrated that in rats IC just the posterior IC subregions are involved in cardiovascular control (Saper 1982; Cechetto 1987; Oppenheimer and Cechetto 1990; Zhang and Oppenheimer 1997). Previous evidence suggests that the IC contributes to autonomic‐ and cardiovascular‐related activity through the lateral hypothalamic area (LHA) (Oppenheimer et al. 1992b) and rostral ventrolateral medulla (Marins et al. 2016). Therefore, enhanced activation of intermediate IC as a consequence of hypertension can contribute to enhanced sympathetic activity under this condition. In agreement, Strazielle and Lalonde (Strazielle et al. 2004) demonstrated that SHM have increased cytochrome oxidase in limbic areas including the intermediate IC and hypothalamus, suggesting that specific regions that modulate sympathetic nerve discharge are activated in SHM, possibly due to mitochondrial dysfunction and excitotoxicity. Supporting NMDA‐mediated higher autonomic and cardiovascular responses in SHR via intermediate IC, we observed increased NMDAR density. These data suggest that NMDAR overexpression in the intermediate IC contributes to increased sympathetic responses.

Increased IC neuronal activity may result in changes in the functional and structural components of the NVU. Supporting activity‐evoked changes in blood vessel density, we observed increased angiogenesis selective to the cardiovascular representative region, intermediate IC, with no concurrent changes in the anterior IC, an area not involved with cardiovascular control. We propose the observed increases in vascular density serves as an adaptive mechanism to support enhanced neuronal and metabolic activity.

Alterations in neuronal activity and vasculature density have been previously associated with concomitant changes to other components of the NVU namely, glial structures (Attwell 1994; Du et al. 2015; Filosa et al. 2016). It is important to mention that IC receives inputs from baroreceptors and other visceral afferents (Cechetto 2014), rasing the possibility that hypertension‐evoked changes to the NVU result from differences in the input.

Astrocytes are essential for the maintenance of cerebral homeostasis; these cells make contact with thousands of synapses and nearby blood vessels (Filosa et al. 2016). In disease conditions including hypertension, astrocytes have been shown to undergo structural changes including gliosis (Tagami et al. 1990; Tang et al. 1993; Yamagata 2012) in the hippocampus (Sabbatini et al. 2000, 2002) frontal cortex, occipital cortex and striatum (Tomassoni et al. 2004a) of SHR, hippocampus, frontal cortex and parietal cortex of hypertensive obese Zucker rats (Tomassoni et al. 2013) and hippocampus and frontal cortex of SHR with diabetes (Tomassoni et al. 2004b). In addition, astrocytes contribute to the stability of the neuronal (Latov et al. 1979; Hatten et al. 1991) and vascular (Filosa et al. 2004; Attwell et al. 2010; Filosa and Iddings 2013) microenvironment (Unger 1998). Consistent with previous findings, we show increased GFAP immunoreactivity and increased astrocyte‐vessel contact within the intermediate IC of SHR. Suarez and colleagues previously showed that interactions between endothelial cells and astrocytes (GFAP) persist until endothelial cells are morphologically differentiated, supporting a role for astrocytes in the regulation of capillary formation (Suarez et al. 1994). Additionally, vascular injury triggers reactive astrogliosis via molecules produced/released at the injury site, driving astrocytes to become activated and promote vascular remodeling (Liberto et al. 2004; Buffo et al. 2010). A possible mechanism contributing to this process is VEGF (vascular endothelial growth factor) secretion by reactive astrocytes after traumatic injury (Winter et al. 1995; Papavassiliou et al. 1997; Rosenstein and Krum 2004) and inflammatory lesions (Argaw et al. 2009). In addition to its neuroprotective effects (Rosenstein and Krum 2004), VEGF strongly promotes angiogenesis (Carmeliet 2005). Thus, in the intermediate IC of SHR increased astrogliosis may be associated with increased angiogenesis.

Increased GFAP expression may result in a physiological processes associated with vascular changes (Lin et al. 1990; Zonta et al. 2003; Xu et al. 2008), such as angiogenesis (Lin et al. 1990; Ritz et al. 2009), or may result in a pathological response such as inflammation (Dong and Benveniste 2001; Farina et al. 2007). To determine whether increased GFAP expression was linked to inflammation in the intermediate IC, we evaluated the morphology of microglia, a key modulator of immune responses in CNS. Microglia respond to brain injury including inflammation (Streit 2000; Shi et al. 2010) and chronic neurodegenerative diseases (Walker and Lue 2005; Perry et al. 2010; Cox et al. 2015; Tang and Le 2016). During the brain inflammatory response, microglia exert neurotoxic functions through the production and release of reactive oxygen species and cytokines (i.e. in ischemic stroke) (Clausen et al. 2008; Thiel and Heiss 2011; Li et al. 2013; Morrison and Filosa 2013; Ritzel et al. 2015). Our data indicate activation of microglia in the intermediate IC of SHR as demonstrated by reduced branch complexity and de‐ramified microglia (Morrison and Filosa 2013). Consistent with our findings, activated microglia have been reported in other central nervous system areas, such as paraventricular nucleus of hypothalamus (Biancardi et al. 2014) and nucleus tractus solitarius (Waki et al. 2011) of hypertensive rats.

The participation of inflammatory components in the brain during hypertension has become a target of several studies. Patients with primary hypertension have increased circulating levels of inflammatory molecules, such as C‐reactive protein, tumor necrosis factor, interleukin 6, and adhesion molecules, such as P‐selectin (Rahman et al. 2002; Koh et al. 2003; Sanz‐Rosa et al. 2005). Intracerebroventricular infusion of minocycline (an anti‐inflammatory/antibiotic that inhibits activation of microglia) in AngII‐induced hypertensive rats promotes decreases in blood pressure, attenuation of cardiac hypertrophy, reduction in plasma norepinephrine levels, attenuation of proinflammatory cytokines IL‐6, IL‐1*β*, TNF‐*α* and elevation of anti‐inflammatory cytokine IL‐10 (Shi et al. 2010). Along the same lines, angiotensin receptor blockers that cross the blood brain barrier are able to decrease macrophage infiltration, normalize the levels of brain endothelial nitric oxide, TNF‐ *α*, IL‐1, and intercellular adhesion molecule (Ando et al. 2004). Numerous studies have demonstrated that inflammatory cytokines have profound effects on neuronal activity (Kang et al. 2008; Colombari et al. 2010) specifically, inflammatory cytokine IL‐1*α* which activates the sympathetic nervous system and increases blood pressure (Kannan et al. 1996; Lu et al. 2009). Thus, it is possible to speculate that increased microglia activity in the intermediate IC contributes to the release of proinflammatory factors that enhance NMDA receptor activation, driving increases in sympathetic output. Importantly, all changes reported were specific to the intermediate IC of SHR and not the anterior IC supporting a selective effect of hypertension in this cardiovascular‐sympathoexcitatory region.

## Perspective and Significance

The current study shows that the intermediate IC of SHR present an increased sympathetic response to NMDA receptor stimulation and inhibition of this receptor have a tonic control in autonomic/cardiovascular control. In addition, the intermediate IC of SHR rats show augmented NMDA receptor density and alterations to the structural components of the NVU. Altogether, our results suggest that increased sympathoexcitation in the SHR can be attributed, in part, to alterations to the NVU within the IC leading to changes in NMDA‐mediated sympathoexcitatory mechanisms/pathways controlled by intermediate IC. Whether these changes in the intermediate IC are a cause or consequence of hypertension in SHR remains to be determined .

## Conflict of Interest

No conflicts of interest, financial or otherwise, are declared by the author(s).
